# Developmental Thresholds and Thermal Requirements of Two Pupal Parasitoids of the Invasive Fall Webworm

**DOI:** 10.3390/insects16030284

**Published:** 2025-03-08

**Authors:** Mustafa Said Bayram, Gülay Kaçar, Luca Rossini, Nuray Baser

**Affiliations:** 1 Department of Plant Protection, Faculty of Agriculture, Bolu Abant Izzet Baysal University, Golkoy, Bolu 14030, Türkiye; saidbayram52@gmail.com; 2 Service d’Automatique et d’Analyse des Systèmes, Université Libre de Bruxelles, 1050 Brussels, Belgium; luca.rossini@ulb.be; 3 International Centre for Advanced Mediterranean Agronomic Studies of Bari (CIHEAM Bari), 70010 Valenzano, Italy

**Keywords:** *Hyphantria cunea*, *Chouioia cunea*, *Psychophagus omnivorus*, thermal performance, development rate functions

## Abstract

The fall webworm is an injurious pest for many host plants in agriculture, forest, and urban environments. Recent observations showed that the faster spread of this pest can be supported by temperature increases, with a subsequent higher severity of the outbreaks and higher impact on the environment. Traditional chemical control is not effective on this pest, while biological control is a valuable alternative, especially if native natural enemies are used. In this study, the performance of Turkish populations of two parasitoids—*Chouioia cunea* and *Psychophagus omnivorus*—at different temperatures is evaluated. Laboratory trials were conducted in climatic chambers under five constant temperatures (10, 15, 20, 25, and 30 °C) and cold storage conditions (6 and 12 °C) for periods of 15, 30, and 45 days. The optimal developmental temperature and the upper thermal threshold of the two parasitoids were similar, while *C. cunea* showed a lower thermal threshold temperature. Both species could develop and survive between 20 and 30 °C, whereas development below 20 °C for *C. cunea*, and below 15 °C for *P. omnivorus* was not possible, respectively. *Chouioia cunae* was less tolerant to temperature changes than *P. omnivorus* in terms of offspring survival, development, reproduction, and parasitism. Under cold storage temperatures, the two species did not develop into adults. This study provided relevant eco-biological information that can further support biological control programmes.

## Simple Summary

The fall webworm, *Hyphantria cunea* (Lepidoptera: Erebidae) is an invasive insect pest that threatens agricultural crops such as fruit trees (especially hazelnut) forests, and urban vegetation areas. Besides the damages to the host plants, the fall webworm also impacts citizens day life. Last instar larvae often enter houses, shops, stores, or vehicles to pupate, leading to allergic skin reactions in sensitive individuals triggered by the long larval hairs. The climate change of recent years is causing extreme temperature conditions, which seems to be more favourable for the development of this pest and possibly of its natural enemies. The effect of temperature, known on this pest, is still poorly explored in relation to its two main hymenopteran parasitoids, *Chouioia cunea* Yang (Eulophidae) and *Psychophagus omnivorus* (Walker) (Pteromalidae). Testing the effect of indigenous parasitoids at different temperature conditions can yield valuable information to plan alternative control strategies for the fall webworm. Laboratory tests conducted at different temperatures are a good starting point to gain insights about the main biological features of the two parasitoid species, such as development, survival, reproduction, and parasitism. This study aims to investigate on this topic, as such information is still scarce in the current literature.

## 1. Introduction

The fall webworm, *Hyphantriacunea* (Drury) (Lepidoptera: Erebidae) is an invasive insect pest that can develop on more than 600 host plant species, such as row crops, herbaceous and fruit plants, ornamental trees, wild shrubs, and forest plants [1,2,3,4,5]. This species is native to North America, but over the last century it has been accidentally introduced in seven Asian and twenty-tree European countries [1,6,7,8]. In Türkiye, the fall webworm was firstly reported in Istanbul (1945); to date, it has spread southwards to 32° N and northwards to 41° N [9]. Nowadays, the fall webworm is considered a threat not only to agricultural and forest environments, but also to urban areas, as infestations in public parks and private gardens are common.

The fast diffusion of this species is supported by different factors, such as the diversity of host plants and morphological and behavioural aspects. An example at hand is the silk web nesting that protects larvae from the attack of natural enemies, retains the heat, and, accordingly, facilitates feeding [5]. The larvae in web nests feed on the leaves, leading to extensive defoliation. Heavy infestations can rapidly result in production losses in agricultural environments and change the visual appearance of landscapes in urban and natural contexts. In fact, larval feeding activity can weaken the structure of the trees, endangering the safety of urban park users [10,11]. In forest environments, the fall webworm can significantly mine the health of the plants with a subsequent perturbation of the ecological niches, especially in newly infested areas [12]. Besides the damage on the vegetation, this pest is a direct issue for humans and domestic animals as well. Larvae often colonise domestic environments or parked vehicles, leaving unpleasant waste. Larval hairs, if touched, can also provoke allergic reactions in people who are susceptible as well as domestic animals, especially dogs.

The invasion of the fall webworm in Türkiye was limited to the northern areas, including the Black Sea and Marmara regions. Mitochondrial (mt) DNA analyses showed that the group responsible for the initial outbreak was a single north American population [13]. In Türkiye, the fall webworm usually produces two generations per year; adults of the overwintering generation appear in early spring, while adults of the first generation of the year occur in mid-summer and produce a second generation in late fall [10,11]. The number of generations, however, depends on the climatic conditions; for instance, in Japan, three generations per year are common [14,15]. The fall webworm overwinters as pupa in cocoons; the last larval stage leaves the nest and chooses tree barks, fences, house roofs, tree hollows, debris at or just below the soil surface, stones, and bark crevices as overwintering locations [15,16,17,18].

If the pest population is below the threshold level, it does not cause considerable damage. Populations can be kept low thanks to many beneficial arthropods, including insect parasitoids, predators, and spiders. The main biological controllers are parasitic wasps and flies, which limit the growth of *H. cunea* populations in both their native and introduced areas, such as Korea, Japan, and Europe [1,19,20]. To date, over 110 parasitoid species have been identified as biological control agents of *H. cunea* preimaginal stages; they include 56 Chalcididae (Hymenoptera) and 54 Tachinidae (Diptera) [20,21]. Among those, one new parasitoid species, *Conomorium metmetsahani* sp. nov. Kaçar&Doğanlar (Hymenoptera: Pteromalidae), has been recently detected in Türkiye [22]. Historically, two Chalcidoidae species, *Chouioiacunea* Yang (Eulophidae) and *Psychophagus omnivorus* (Walker) (Pteromalidae), are known for their efficacy in reducing fall webworm populations [16,21], attracting the interest of scholars and institutions. These parasitoids are gregarious endoparasites of *H. cunea* pupae. Adults lay multiple eggs on a single larval host, where they develop for the overall duration of the preimaginal stage [23,24]. Previous studies highlighted that *P. omnivorus* parasitised 6.3–8% of the overwintered *H. cunea* pupae in Samsun province of Türkiye [25,26]. In the same area, *P. omnivorus* and *C. cunea* parasitised 1.9% and 6.7% of the pupae and represented 11% and 79% of Chalcididae rearing, respectively [21]. In Georgia, *C. cunea* was primarily responsible for the mortality of 70–80% of overwintering pupae of *H. cunea* [27].

Although *C. cunea* is a generalist parasitoid, its parasitisation rate showed a remarkable effectiveness on *H. cunea* (68.2–83.2%) [28]. Accordingly, it was mass-reared and effectively involved in biological control programmes conducted in China [24,29,30]. These encouraging outcomes show that the combination of suitable mass-rearing and release methods is fundamental for successful control strategies.

Natural enemies are subject to the same living conditions as their hosts because of parasitoid–host co-evolution and olfactory cues. Accordingly, an evaluation of their response to environmental changes is pivotal for further planning mass releases and to identify the natural enemy that is more suitable for a given area. As insects are ectotherms, temperature can be considered the most limiting ecological factor for their development. Currently, the knowledge of the thermal response of *C. cunea* and *P. omnivorus* in terms of development, survival, fertility, and parasitisation rate is still limited, leading IPM scientists and technicians to make decisions on an empirical basis.

Given the importance of this information, this study aimed to assess the thermal performance of *C. cunea* and *P. omnivorus* Turkish populations. The effect of temperature on five main biological parameters (survival, development, reproduction, fecundity, mortality, and parasitism) was explored at a wide range of constant temperatures. The second part of this study, instead, explored the effect of storage times of 15, 30, and 45 days at low temperatures (6 and 12 °C) for their possible role in mass production for biological control.

## 2. Materials and Methods

### 2.1. Insect Colonies Rearing

Previous studies ascertained that *C. cunea* and *P. omnivorus* could be easily reared in large quantities using *Galleria mellonella* (L.) (Lep.: Galleridae) as a host [31,32]. Our experiments were conducted at the University of Bolu Abant Izzet Baysal in Bolu, Türkiye. *C. cunea* and *P. omnivorus* were collected using cardboard bands from infested overwintering *H. cunea* pupae in walnut orchards in Düzce, Türkiye. Continuous laboratory rearing of the parasitoids was further maintained for pupae of *G. mellonella* placed in climatic chambers (Nuve Laboratory & Sterilization Technology, Ankara, Türkiye; Model TK600) set at 23 ± 1 °C, 60–70% RH, 16:8 h [L:D]. The starting colony of *G. mellonella* was collected from honeybee producers in Bolu and subsequently reared in small cages (10 cm × 10 cm × 10 cm) placed in climatic chambers set at 28 ± 2 °C, 60% RH, 16:8 [L:D]; specimens were fed an artificial diet according to [33]. Continuous rearing of the three populations were periodically refreshed by collecting, in spring and/or fall, 50–100 individuals of each species from the field. The wild specimens were subsequently introduced into the growth chambers, ensuring good vigour and a wider genetic diversity.

As the parasitoids depend on their host, the continuous rearing was organised as follows. The adults of the two parasitoid species were separately placed in mesh-screened cages (30 × 30 × 30 cm) containing a 20% honey–water mixture to ensure nutrients and hydration. The 2- to 3-day-old *G. mellonella* pupae were exposed to adult parasitoids for 2–3 days. The parasitoid-exposed pupae were transferred to new cages and placed in growth chambers under controlled conditions until the emergence of adult parasitoids (20 days, circa) [11]. Newly emerged female and male wasps were subsequently placed in 8 × 11 × 14 cm screen cages and fed with a mixture composed of 50% honey–water. The wasps were held for 3–4 days to allow mating and egg maturation before their use in any trial. Tests involved 2–3-day-old *G. mellonella* pupae and 4- to 6-day-old adult female parasitoids and were organised in growth chambers set to specific temperatures.

### 2.2. Effect of Five Constant Temperatures

The effects of temperature on development, survival, sex ratio, and parasitism of *C. cunea* and *P. omnivorus* were evaluated at five fixed temperatures (10, 15, 20, 25, and 30 °C) with 60–70% RH and photoperiod of 16:8 h (L:D). The temperature range chosen for this trial is in line with the average daily minimum and maximum temperatures in a large portion of the Düzce and Sakarya provinces over the period May–October, when *H. cunea* and the two parasitoids are usually active in the orchards [10,11,20,34]. A water container was placed inside each growth chamber to ensure the relative humidity level of 60–70%. Every parasitoid rearing was subject to the same test protocol at each temperature condition.

Ten *G. mellonella* pupae were placed in a Petri dish (1.5 cm in height and 9 cm diameter) and exposed to a female wasp for 72 h to produce parasitised hosts for each replicate under the above-mentioned laboratory settings. Ten repetitions plus an untreated control (where ten host pupae were not exposed to parasitoids) were considered for each temperature treatment and for each parasitoid–temperature combination. Host pupae were placed on wet tissue paper; the female parasitoid was fed and hydrated through a thin line of 40–50% honey–water mixture left on the Petri dish’s edge. Every two to three days, water droplets were sprayed on the tissue to avoid parasitised host pupae drying out. Following the initial emergence of parasitoids, the Petri dishes were systematically monitored twice daily—once in the early morning and once in the late afternoon. Once adult parasitoid emergence had ceased, host pupae were examined under a microscope to identify any fly or parasitoid cadavers. To quantify the total number of adult parasitoids that emerged, developmental times (from egg to adult) were aggregated across the overall replicates and temperature treatments.

### 2.3. Cold Storage of Chouioia Cunea and Psychophagus Omnivorus

Parasitised pupae were kept at 6 °C and 12 °C for 15, 30, and 45 days to compare cold storage between *C. cunea* and *P. omnivorus*. Five *G. mellonella* pupae were exposed to a female wasp in a Petri dish (same conditions as in Section 2.2) for 72 h. The exposed hosts were subsequently transferred to either 6 °C or 12 °C. Ten repetitions were considered for the two parasitoids’ experimental trials. After exposure to the cold condition, host pupae were held in growth chambers at 25 °C, a 16:8 h (L:D) photoperiod, and 60–70% RH. Specimens were monitored daily to assess the adults’ emergence; the sex and developmental time of the emerged wasps were noted down. Five untreated control repetitions (where five host pupae were not exposed to parasitoids) were included in each parasitoid–temperature combination. The host pupae were dissected to check the appearance of adult flies or wasps.

### 2.4. Data Analysis

Linear and nonlinear regression models [35] were considered to estimate the development rate *D*(*T*), thermal thresholds, and degree-day (*DD*) requirements for male and female insects. These models have been widely applied to extract quantitative information from life tables data and insect population modelling [36,37].

The linear model assumes a direct relationship between temperature and development rate:

(1) $$D\left(T\right)=a+bT$$

where *D*(*T*) is the development rate (1/day) at temperature *T*, and a and b parameters with the following biological meaning. The minimum temperature threshold $T_{b}$, below which development is theoretically not possible, can be calculated as:

(2) $$T_{b}=-\frac{a}{b}$$

Similarly, the degree-day (*DD*) requirement for development can be calculated as:

(3) $$DD=\frac{1}{b}$$

The linear model (1) is commonly used for estimating the thermal requirements, in terms of degree days, of insects [38], but it neither accounts for nonlinearities at extreme temperatures nor clearly defines the optimal temperature for the development.

For this reason, this study considers the Brière model as well, a second well-known development rate function that better describes the typical increasing–decreasing trend of life table datasets. Additionally, the Brière equation contains parameters that describe quantitative information about the species. From a mathematical point of view, the Brière model [35] is presented as:

(4) $$D\left(T\right)=nT\left(T-T_{b}\right){\left(T_{L}-T\right)}^{\frac{1}{m}}$$

where $T_{b}$ and $T_{L}$ are the lower and upper thermal thresholds below and above which development is theoretically not possible, respectively, and *n* and *m* are empirical parameters with no biological meaning.

As the Brière function provided the thermal thresholds, we could compute more accurately the degree-day (*DD*) accumulation required for complete development, as in [39]:

(5) $$DD=\sum_{i=1}^{n}\left(T_{i}-T_{b}\right)\Delta t$$

where $T_{i}$ is the mean daily temperature, $T_{b}$ is the minimum thermal threshold, and Δt=1 is the integration step (usually set to one day). It is worth remarking that if $T_{i}<T_{b}$, no development occurs. This method is widely used for the phenological modelling of insect populations [40].

#### Model Performance, Goodness-of-Fit Evaluation, and Data Analysis

The best fit parameters of Equations (1) and (4) were estimated through a least-square regression. Fitting performances were assessed, as in [41,42,43], through the coefficient of determination $R^{2}$. All the calculations of this part of the study were carried out using the ad hoc Python (vers. 3.13) script available at https://github.com/lucaros1190/HcuneaParasitoids (accessed on 3 March 2025).

The raw dataset, instead, was analysed to check statistical differences between the parasitoid–temperature combinations, as well. The development time, mortality, fecundity, number of emerging females and males, and parasitism of *C. cunea* and *P. omnivorus* at the different temperatures were summarised by computing the means and standard errors.

Before the analysis, data were checked for normality through the Shapiro–Wilk test. Significant differences (*α* < 0.05) between different temperatures were assessed, as follows. The differences in terms of development time between the species and the two sexes were assessed through a linear model (LM) followed by Tukey’s test *post hoc,* considering the development time as response variable and temperature and sex as factors. Lifetime fecundity per female (eggs), larval and pupal mortality, and adults’ emergence were analysed through the Kruskal–Wallis (KW) test followed by Dunn’s *post hoc*. The offspring produced by each female, instead, were assessed through a generalised linear model (GLM) followed by the Bonferroni (*p* < 0.05) test *post hoc*, considering the offspring produced as a response variable and temperature and sex as a factor. All the calculations of this study were carried out using RStudio software (vers. 4.4.2) (R Core Team).

## 3. Results

### 3.1. Effect of Constant Temperatures

**Figure 1:**
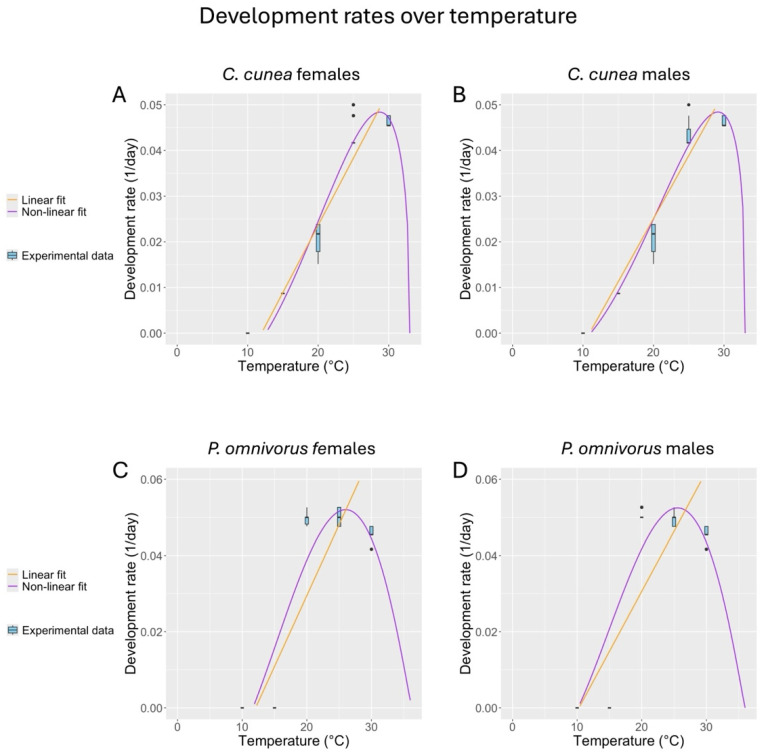
Best linear (orange lines) and Briére (purple lines) fit curves for males (**A**,**C**) and females (**B**,**D**) *C. cunea* and *P. omnivorus*, respectively. The best fit parameters are listed in Table 2.

The temperature-dependent development rates of *C. cunea* and *P. omnivorus* were well described by the linear and the Briére model (Figure 1), although e data were better represented by the Briére model. The two functions, combined with the synthetic values reported in Table 1, provided relevant information on the thermal response of both species. *C. cunea* could develop at 15–30 °C but not below 15 °C. *P. omnivorus* successfully developed at 20–30 °C but no development was observed below 20 °C. The lower threshold temperature, accordingly, was higher than that of *C. cunea*, whereas the optimal and upper temperatures for the development of both species were comparable According to the Brière model, the optimal temperature for the development of males and females of *C. cunea* and *P. omnivorus* is 30 ± 5 and 30 ± 1 °C, respectively.

No statistical differences were assessed in terms of development times between the two species, for females (LM, *t* = −0.34, *p* = 0.743) and males (LM, *t* = −0.34, *p* = 0.742) (Table 1). The thermal requirements to complete the development for females and males were 340.1 and 268.8 degree-days (*DD*) for *C. cunea*, 362.3 and 317.4 for *P. omnivorus* (Table 2).

The number of hosts parasitised by *C. cunea* was not affected by temperature (KW, χ^2^ = 6.103, *df* = 4, *p* = 0.192), while the parasitism of *P. omnivorous* increased at 20–30 °C (KW, χ^2^ = 36.436, *df* = 4, *p* < 0.05). Temperature had no significant effect on the larval and pupal mortality of both species (KW, *C. cunea*, χ^2^ = 2.182, *df* = 4, *p* = 0.702; *P. omnivorus*, χ^2^ = 3.063, *df* = 4, *p* = 0.547). *P. omnivorius* produces more eggs than *C. cunea* only at 25 °C (Table 3); the opposite was observed for the other rearing conditions. Temperature significantly affected the emergence of adults of *C. cunea* (KW, χ^2^ = 10.44, *df* = 4, *p* < 0.05) and *P. omnivorus* (KW, χ^2^ = 37.63, *df* = 4, *p* < 0.05) (Table 3).

The sex ratio of the two parasitoid species was affected by temperature (Table 4), as assessed by the GLM under all temperature conditions, *C. cunea* produced more females than *P. omnivorus*. The female/male sex ratios for *P. omivorus* and *C. cunea* were found between 0.15 and 0.27 and 38.77–59.22, respectively (Table 4).

**Figure 2:**
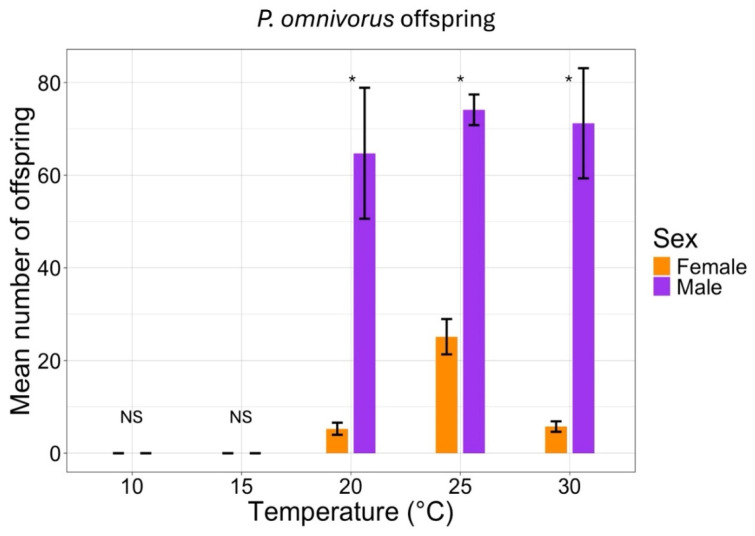
Effect of temperature at 10–30 °C with a photoperiod of 14:10 (L:D) h on the offspring sex ratio (number of females and males) of emerging adults of *P. omnivorus*. The star (*) and ‘NS’ above the bars indicates significant and not-significant differences (*p* < 0.05) according to the analysis described in Section 2.4, respectively.

**Figure 3:**
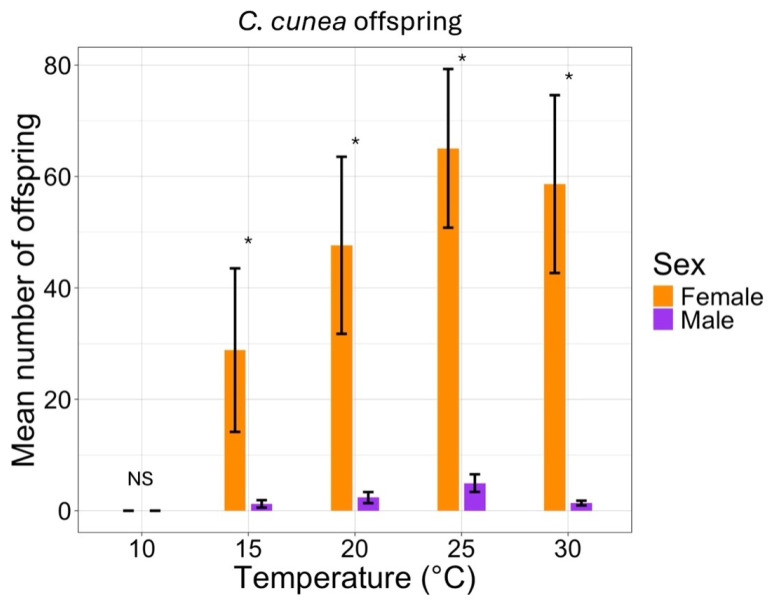
Effect of temperature at 10–30 °C with a photoperiod of 14:10 (L:D) h on the offspring sex ratio (number of females and males) of emerging adults of *C. cunea*. The asterisk (*) and ‘NS’ above the bars indicate significant and not-significant differences (*p* < 0.05) according to the analysis described in Section 2.4.

The percentage of males that emerged from the offspring was affected by temperature and was significantly different for the two species (Figure 2 and Figure 3). A similar scenario was observed for females, but fewer females were produced by *P. omnivorus* (Figure 2 and Figure 3).

### 3.2. Cold Storage

There was no successful development into adults when both species were placed in cold storage at 6 and 12 °C for 15, 30, and 45 days. The eggs or larvae of *C. cunea* and *P. omnivorus* did not complete their development unless they were removed from cold storage.

## 4. Discussion

This study explored the thermal performance of *Chouioia cunea* and *Psychophagus omnivorus*, two parasitoids of *Hyphantria cunea*. This piece of knowledge is fundamental for further identification and application of biological control techniques based on resident natural enemies [44]. Local natural enemies are crucial to pest management of alien species, as they avoid the introduction of other natural enemies from native areas and are already well-adapted to the climate conditions [44].

*Chouioia cunea* and *P. omnivorus* are two gregarious endo-parasitoids active on many pest species in Türkiye [21,25,27,45,46]. These two parasitoids are of worldwide interest, as shown by the current literature. A recent work, for instance, identified *C. cunea* as the most successful pupal parasitoid of the fall webworm in China, also suggested it as a promising biological control agent [47]. *Psychophagus omnivorous* was also reported as a suitable candidate for biological control of the fall webworm [21], but the current literature includes only a small number of investigations conducted on this species [21,23,25,26,48]. This study provided helpful insights and added a piece of knowledge on the thermal response of these two species in Türkiye, an environment where this information was still missing.

*Chouioia cunea* can survive, develop, and reproduce in a wider range of temperatures than *P. omnivorus*. The results of this study are in line with the climate conditions of northern Türkiye, where the two species are widely diffused and from where the wild types of *H. cunea* involved in the rearing trials have been collected (Black and Marmara regions) [21,25,26]. The broader thermal tolerance of *C. cunea* likely facilitates its establishment and persistence across a wider geographical range, supporting the distribution observed in these regions.

*Chouioia cunea* showed a significantly higher proportion and total number of female progeny than *P. ominorus*. Conversely, *P. omnivorus* showed a slightly higher fecundity, parasitism rate, and adult emergence, with a higher proportion of male progeny. According to [49] *C. cunea* had a female-to-male ratio ranging from 45:1 to 96:1, with an average clutch size of eggs. Similarly, Ref. [21] reported a female-to-male ratio and average clutch size of 44.5:1 and 117 for *C. cunea*, and 0.92:1 and 60 for *P. omnivorus*, respectively.

Temperature is among the main limiting environmental factors for insects [50]. An interesting result of this study is that *P. omnivorus* could not develop below 20 °C but it was successful between 20 and 30 °C. Conversely, *C. cunea* developed in the range 15–30 °C, but many eggs and larvae failed to turn into adults. Previous studies, such as [51], reported that adult females can live on average 15 days at 21 °C. Survival was comparatively better for *C. cunea* at the lowest temperature investigated, whereas survival of *P. omnivorus* was better at the highest ones.

The developmental time of both species was inversely related to temperature, as expected, being shorter at higher temperatures and longer at lower temperatures. At 25 °C, *C. cunea* completed its development in 13.8 days, which was shorter than the 20 days required by *P. omnivorus*. These findings are consistent with the results of [23], where the developmental duration of *P. omnivorus* at 25 °C ranged from 21.3 to 24.3 days, with variations that depended on the host species. Additionally, Ref. [30] reported that the developmental times for *C. cunea* at 25–26 °C and 70–80% relative humidity were approximately 1.7 days for eggs, 8 days for larvae, and 6 days for pupae. The knowledge of thermal performance of resident natural enemies will support the identification of their geographical gaps and the implementation of potential future biological control strategies [52]. Moreover, the analysis of the effect of cold temperatures on the eggs and larvae of the two parasitoid species provided a clearer idea of the storage conditions required in the case of mass rearing prior to release into the environment.

One of the key factors affecting parasitoid performance from a biological control point of view is the percentage of hosts that are parasitised, often measured by exposing a limitless number of hosts (*ad libitum*) to parasitoids [53]. In this study, the parasitism rate of *C. cunea* remained unchanged across different temperatures, even if *P. omnivorus* showed a slight increase at 25 °C. Parasitism, survival, and reproduction of emerging adults of *C. cunea* and *P. omnivorus* were adversely affected by exposure to both low and high temperatures. For instance, from experiments carried out in China, *C. cunea,* showed a parasitisation rate of 67.74% on average, with peaks of 83.2% [24,28,54] and average clutch sizes of 117, circa [21].

A high performance has been recorded for *P. omnivorus* by studies carried out in Türkiye as well, where it was ascertained a 78.9% parasitisation rate with a clutch size of 60 eggs. A different study, instead, reported *P. omnivorus* clutch sizes ranging from 16 to 47 eggs [26]. In the three host species tested, the proportion of pupae selected for parasitism by *P. omnivorus* within each age group was highly variable, and the wasps did not show a significant preference for any of the pupae of different ages that have been exposed [23]. In our experiments, both species generally lay all their eggs in a single host pupa. Each female of the parasitoid can typically kill one fall webworm, and a single adult of *C. cunea* can typically lay all her eggs in a single fall webworm pupa [29]. Unlike our study, Ref. [23] observed that *P. omnivorus* attacked 1.9–6.9 pupae according to three lepidopteran species.

The survival and reproduction of emerging adults of *C. cunea* and *P. omnivorus* were adversely affected by exposure to low temperatures. We may suppose that the resistance of parasitoids to cold temperatures depends on their developmental stage; the young stages have less probability of survival, and they might need to grow before overwintering. These findings are mostly in line with earlier research carried out on other species that explored the effect of exposure to cold temperatures on parasitoid fitness [50,51,52,53,55,56]. Young stages of both parasitoids appear to be particularly vulnerable to severe temperatures because their overall embryonic development takes place inside the host pupae. The parasitoids’ tolerance to cold temperatures depends on their developmental stage, with pupae or pre-adults having a higher survival rate [52]. Survival rates of different parasitoids in cold storage were shown to decrease over time, with the severity of the effect depending on the species and even the population [50]. Low temperatures are especially harmful to reproductive organs [55]. Also, according to [53], low temperatures can delay the maturation of eggs or cause malformations in the insects’ reproductive organs, with a possible impact on fecundity and subsequent decrease in parasitoids’ fertility after a period of cold storage.

## 5. Conclusions

This study analysed the thermal response of *C. cunea* and *P. omnivorus*, showing that low temperatures are likely a major limiting factor for the establishment and persistence of this biocontrol agent. The exposure to low temperature negatively affected the storage, survival, and reproduction of emerging *C. cunea* and *P. omnivorus,* but we can suggest that the parasitoids’ tolerance can be further investigated in their older stages, such as pupae or pre adults. Although both species were shown as potential biological agents, *P. omnivorus* produced more males in the laboratory. Therefore, *C. cunea* was identified as the most suitable candidate for the control of the fall webworm *H. cunea*.

## Figures and Tables

**Table 1:** Female and male mean developmental time (days ± SE, *n* = 50) from egg to adult emergence of *C. cunea* and *P. omnivorus* at various temperatures.

	*C. cunea*		*P. omnivorus*	
Temp. (°C)	Females	Males	Females	Males
10	--	--	--	--
15	115 ± 0	115 ± 0	--	--
20	48.8 ± 0.3	48 ± 2	19.76 ± 0.06	19.77 ± 0.02
25	23.13 ± 0.04	23.1 ± 0.3	19.98 ± 0.04	20.08 ± 0.02
30	21.50 ± 0.02	21.6 ± 0.1	22.1 ± 0.1	22.06 ± 0.05

**Table 2:** Estimates of the lower (*Tb*) and upper (*T_L_*) temperature thresholds (°C), and degree day (*DD*) requirements for the development from egg to adult emergence of *C. cunea* and *P. omnivorus* using linear and nonlinear models. The table lists the best fit values estimated for both functions, graphically represented in Figure 1.

		Linear Model					Brière Model				
Species	Sex	*T_b_*	*DD*	*a* (·10^−2^)	*b* (·10^−3^)	*r^2^*	*T_b_*	*T_L_*	*n* (·10^–5^)	*m*	*r^2^*
*C. cunea*	Female	11.97	340.1	−3.52 ± 0.05	2.94 ± 0.02	0.867	12.57 ± 0.08	33.0 ± 0.3	5.74 ± 0.43	2.4 ± 0.2	0.952
	Male	10.6	362.3	−3.01 ± 0.02	2.76 ± 0.08	0.930	11.1 ± 0.4	33 ± 2	6 ± 2	3 ± 1	0.966
*P. omnivorus*	Female	9.7	268.8	−4.49 ± 0.02	3.37 ± 0.01	0.624	11.6 ± 0.2	36 ± 1	1.1 ± 0.5	0.9 ± 0.1	0.869
	Male	9.6	317.4	−3.24 ± 0.02	3.15 ± 0.09	0.694	10.1 ± 0.2	36.0 ± 0.6	1.0 ± 0.2	0.92 ± 0.06	0.753

**Table 3:** Means ± SE of larval/pupal mortality, lifetime fecundity, adult emergence, and parasitism of *C. cunea* and *P. omnivorus* at various temperatures (*n* = 50). Different letters in each column indicate significant differences (*p* < 0.05) according to the analysis described in Section 2.4; groups without significant differences are not labelled with letters.

	*C. cunea*				*P. omnivorus*			
Temp. (°C)	Larval/ Pupal Mortality	Lifetime Fecundity per Female (Eggs)	Adults’ Emergence	% Parasitism	Larval/ Pupal Mortality	Lifetime Fecundity per Female (Eggs)	Adults’ Emergence	% Parasitism
10	--	--	--	--	--	--	--	--
15	15 ± 15	60 ± 30	40 ± 20	40	--	--	--	--
20	8 ± 8	70 ± 20	70 ± 20	60	7 ± 7	60 ± 10 b	60 ± 10 b	70
25	10 ± 10	110 ± 30	100 ± 30	70	0 ± 0	120 ± 6 a	120 ± 6 a	100
30	22 ± 12	90 ± 10	70.00 ± 20	90	6 ± 6	70 ± 8 b	60 ± 10 b	80

**Table 4:** Means (±SE) of male and female numbers and of sex ratio (F/M) of *C. cunea* and *P. omnivorus* at various constant temperatures (±0.5 °C).

	*C. cunea*			*P. omnivorus*		
Temp. (°C)	Males	Females	Sexratio (F/M)	Males	Females	Sexratio (F/M)
10	--	--	--	--	--	--
15	1.0 ± 0.6 (5)	40 ± 20 (198)	40 ± 10	--	--	--
20	1.7 ± 0.6 (5)	60 ± 20 (186)	39 ± 3	50 ± 10 (81)	7 ± 2 (15)	0.15 ± 0.02
25	2.3 ± 0.7 (6)	100 ± 30 (263)	46 ± 4	94 ± 4 (110)	26 ± 3 (42)	0.27 ± 0.02
30	1.2 ± 0.4 (3)	70 ± 20 (140)	60 ± 7	52 ± 9 (74)	9 ± 2 (15)	0.27 ± 0.03

## Data Availability

The script and the dataset to fully reproduce the results of this study are publicly available at https://github.com/lucaros1190/HcuneaParasitoids (accessed on 3 March 2025).
